# Food Safety and Bioavailability Evaluations of Four Vegetables Grown in the Highly Arsenic-Contaminated Soils on the Guandu Plain of Northern Taiwan

**DOI:** 10.3390/ijerph110404091

**Published:** 2014-04-14

**Authors:** Shaw-Wei Su, Chun-Chih Tsui, Hung-Yu Lai, Zueng-Sang Chen

**Affiliations:** 1 Department of Agricultural Chemistry, National Taiwan University, Taipei 10617, Taiwan; E-Mails: f94623001@ntu.edu.tw (S.-W.S.); d91623403@ntu.edu.tw (C.-C.T.); 2 Department of Post-Modern Agriculture, MingDao University, Changhua 52345, Taiwan; E-Mail: soil.lai@mdu.edu.tw

**Keywords:** arsenic (As), soil testing, vegetables, phytotoxicity, provisional tolerance weekly intake (PTWI), extraction method

## Abstract

Arsenic contamination in a large area of agricultural fields on the Guandu Plain of northern Taiwan was confirmed in a survey conducted in 2006, but research concerning the relationship between bioavailable As concentrations in contaminated soils and crop production in Taiwan is not available. Pot experiments were conducted to examine the growth and accumulation of As in four vegetable crops grown in As-contaminated soils and to assess As intake through consumption. The phytotoxic effects of As in soils were not shown in the pot experiments in which vegetable crops were grown in soils contaminated with different As levels *in situ* collected from Guandu Plain (120–460 mg/kg) or artificially spiked As-contaminated soils (50–170 mg/kg). Experimental results showed that the bioavailable As extracted with 5 M NaHCO_3_ from soils can be used to estimate As concentrations in vegetables. The As concentrations in the vegetables were compared with data shown in the literature and As limits calculated from drinking water standards and the provisional tolerance weekly intake (PTWI) of inorganic As established by the Food and Agriculture Organization of the United Nations/World Health Organization (FAO/WHO). Although the As levels in the vegetables were not high and the bioavailability of As in the soils was quite low, long-term consumption may result in higher As intake in the human body.

## 1. Introduction

Arsenic (As) is an environmental contaminant that is a problem worldwide because this element is found in many fields and it is toxic to plants, animals, and humans [1,2,3]. As is toxic to plants because the chloroplast membrane and cell membrane are damaged by peroxidation of the membrane lipid [4]. The symptoms of As toxicity in plants have been variously described as wilting leaves, violet coloration (due to increased anthocyanin levels), root discoloration, inhibition of root growth, cell plasmolysis, and plant death [5,6]. Since there is evidence that As can cause cancer in humans, the International Agency for Cancer Research has classified As as a group 1 carcinogen. In soils, As presents as oxyanion forms similar to phosphorus (P) [7] and occurs mainly as an inorganic species [8]. In well-aerated soils, arsenate [As(V)] is the predominant form, whereas in a reduced environment such as paddy soils, the arsenite [As(III)] species prevails. Arsenic may accumulate in plants and animals, and eventually be transferred to humans through the food chain [9].

Arsenic is easily immobilized in soil, and thus As bioavailability in and phytotoxicity to crops can be alleviated [2,10]. However, several studies found that As concentrations in crops can reach levels that are unsafe for human health. For example, Bhumbla and Keefer [11] reported high As concentrations (6–12 mg/kg FW) in alfalfa cultivated in low As-contaminated soils (total As 25–50 mg/kg). Gulz *et al.* [12] indicated that As transport from root to shoot is significant in sunflower, English ryegrass, and rape cultivated in As-spiked soils, and As concentrations in the rape leaves reached 15–37 mg/kg. In some cases, the phytotoxic effects of As cannot ensure the food safety of crops produced in As-contaminated fields. Accordingly, predicting As levels in crops based on labile concentration in the corresponding soils is important. In addition to consuming crops, drinking groundwater is also another exposure pathway of As intake by humans in As-contaminated areas [13,14,15].

The bioavailable As concentration in soils for crops can be determined using chemical extraction agents. As concentrations in crops can be predicted based on the specific relationship between extractable As levels in soils and crops. This technique can be used to predict the potential risks of food safety before a crop is cultivated. However, only limited published studies have performed suitable testing protocols for As bioavailability in soils. For example, Woolson *et al.* [16] compared six soil testing methods for predicting As levels in maize that was grown in 28 soils in eight states in the United States. The researchers recommended the reagent 0.5 N NaHCO_3_ or mixed acids 0.05 N HCl and 0.025 N H_2_SO_4_. Peryea [17] reported that As levels in apple leaves and fruit were well correlated with soil As extracted with 0.5 N NaHCO_3_ or an aqueous solution of glacial acetic acid. Field studies in China found a significant correlation between As levels in brown rice and vegetables and in soils extracted with 0.005 M DTPA-TEA (pH 7.3) or 0.5 M NaH_2_PO_4_ [18,19]. Many studies also indicated that soil As extracted with 0.05 M (NH_4_)_2_SO_4_ and 0.05 M (NH_4_)H_2_PO_4_ can represent the bioavailability of As in soil or the potential As available in soil [20,21,22,23].

As contamination in a large area of agricultural fields on the Guandu Plain of northern Taiwan was confirmed in a survey conducted in 2006 [24]. According to the survey results, about 128 ha of rice soils were seriously contaminated with As (higher than 60 mg/kg). Approximately 60%–85% of the As on the surface 0–60 cm were present in the clay fractions [25]; the source of As in this area is the hot spring water from the geothermal spring located roughly 5 km northeast of the Guandu Plain. The hot spring water mixes with stream water that has been used for irrigation for decades. Four decades ago, use of the As-tainted stream water as irrigation water ceased. The survey also reported that the cultivated vegetable crops in the fields showed no evidence of toxicity, and the As concentrations in the collected vegetable crop samples were lower than the detection limit (1.0 mg/kg DW). However, there were no descriptions of how the growth condition of the crops was determined. The reported normal growth of crops seemed to rely simply on observation without discussion of crop yields. To understand the effects of As-contaminated soils on crop growth, a comparison of crops produced in areas with different levels of As contamination in soils is required. In addition, the detection limit for As in crops reported in the previous survey is too high compared with that reported in other studies [13,14,26]. To assess the food safety of crops grown in As-contaminated fields on the Guandu Plain, a reliable technique for measuring the As concentrations in crops must be used.

Since the previous field survey focused on measuring total As concentrations in soils on the Guandu Plain, the limited data for vegetable crops collected randomly from the fields were not reliable to assure the safety of the crops for consumers. Pot experiments in a greenhouse can overcome large variations in weather and soil conditions in the field and control the factors under investigation. A rigorous study using pot experiments is required to assess the potential risks of As-contaminated soils on the Guandu Plain in agricultural production. The objectives of this study are: (1) to examine the negative effects of As in soils on crop growth; (2) to understand the accumulation of As in vegetable crops grown in intrinsic or artificial As-contaminated soils and assess food safety; and (3) to compare different soil testing methods for predicting As levels in vegetable crops.

## 2. Materials and Methods

### 2.1. Selection of Vegetable Crops

The vegetable crops used in this study include carrot (*Daucus carota* L.), garland chrysanthemum (*Chrysanthemum coronarium*), loose head lettuce (*Lactuca sativa* L. var. longifolia), and loose leaf lettuce (*Lactuca sativa*). These vegetables are generally cultivated in As-contaminated fields on the Guandu Plain and are common vegetables consumed by Taiwanese. Adriano [27] indicated that carrot is better at enduring As toxicity than other edible crops. It is possible that As can accumulate in the edible part of the carrot without displaying toxicity symptoms. Local inhabitants also doubt that the edible carrot root grown in the As-contaminated soil may absorb more As than leaf vegetables. The long growth period (three months) may also increase the possibility of As accumulation in carrot roots. Lettuce was selected as the representative leaf vegetable because lettuce is commonly studied in the literature. Two kinds of lettuce were cultivated in this study: loose head lettuce and loose leaf lettuce. Garland chrysanthemum is a common winter vegetable generally used for hot pot, a cooking method popular in Taiwan. The growth period is as long as two months, which is longer than lettuce (40 days), and this long growth period may provide more opportunities for garland chrysanthemum to accumulate As in edible parts.

### 2.2. Soil Sampling

Four soil samples containing different levels of As (Ck for control soil, and L, M, and H for soils with low (L), medium (M), and high (H) As concentrations, respectively, were used to test the effects of As on the growth of three crops. Approximately 20 kg for each concentration were collected from the fields on the Guandu Plain for the carrot, garland chrysanthemum, and loose head lettuce pot experiments. The total As concentration for Ck, L, M, and H was approximately 20, 120, 200, and 270 mg/kg, respectively. To increase the differences in the total As concentration, an extra soil sample with a higher level (2H) of total As (450 mg/kg) was collected from the agricultural field on the Guandu Plain to replace the H soil for the pot experiment of loose head lettuce. The soil samples were air-dried, ground by hand using a woody hammer, sieved to <1.6 cm, and mixed homogeneously for the pot experiments. A small part of the soil samples were further ground by hand using a woody bar, sieved to <2 mm, and stored in plastic bottles for additional laboratory analysis.

To further increase As bioavailability in soils and investigate the effects on crop growth, artificial As-contaminated soils were also prepared for the loose leaf lettuce pot experiment. Another four soil samples containing different levels of As (denoted as Ck2, L2, M2, and H2, 20 kg soils for each treatment) in the fields on the Guandu Plain were collected. In addition to the *in situ* As-contaminated soils (L2, M2, and H2), an additional 100 kg of the Ck2 soil sample was collected to prepare artificial As-contaminated soils as described in Section 2.3.

### 2.3. Artificially Spiked to Change the Bioavailability of As

The additional 100 kg of Ck2 soil sample collected *in situ* in the As-contaminated site was divided into five equal portions (20 kg for each portion). Na_2_HAsO_4_·7H_2_O solution was added to each portion to increase the total As soil concentration 30, 60, 90, 120, and 150 mg/kg, respectively (denoted as Ck2 + 30, Ck2 + 60, Ck2 + 90, Ck2 + 120, and Ck2 + 150, respectively). The artificially As-spiked soil samples were incubated for 30 days, and the soil water content was maintained at 65% of the water-holding capacity (WHC) of the soil samples by weighing and adding deionized water every three days. During the incubation period, each As-spiked soil sample was thoroughly stirred with a shovel every week. After the incubation period, all soil samples were air-dried, ground by hand using a woody hammer, sieved to <1.6 cm, and mixed homogeneously for the pot experiments. A small part of the soil samples was further ground by hand using a woody bar, sieved to <2 mm, and stored in plastic bottles for further laboratory analysis.

### 2.4. Soil Analysis

The soil pH was analyzed using a glass electrode in mixtures of soil and deionized water (*w/v* = 1:1) [28]. The total As concentrations of the soils were determined with the HNO_3_/H_2_O_2_ digestion method [29]. The bioavailable As concentrations were extracted with the following six methods: (i) 0.5 M NaHCO_3_ [16]; (ii) 0.05 N HCl + 0.025 N H_2_SO_4_ mixed acid solution [16]; (iii) 0.005 M DTPA-TEA (pH = 7.3) [18]; (iv) 0.05 M (NH_4_)_2_SO_4_ [20]; (v) 0.05 M (NH_4_)H_2_PO_4_ [20]; and (vi) 0.5 M NaH_2_PO_4_ [19].

The As concentrations in all digested and extracted solutions were determined with a hydride generation atomic absorption spectrometer (PerkinElmer AAnalyst 200 fitted with a flow injection system, FIAS 400, PerkinElmer, Waltham, MA, US). The NIST SRM 2710 Montana soil (certified As concentration: 626 ± 38 mg/kg) was used as the certified reference material (CRM) for quality control of the laboratory analysis. The average total As recovery percentage with the HNO_3_/H_2_O_2_ digestion method was 94 ± 5% for this CRM.

### 2.5. Pot Experiments

All soil samples (3.0 kg of the dried soil) were put in a Wagner 1/5000a pot (soil depth = 15 cm). During the pot experiments, the water content of potted soils under different treatments was maintained at approximately 65% of the WHC. The four vegetables were cultivated in the following order: carrot, garland chrysanthemum, loose head lettuce, and loose leaf lettuce. Ten seeds for each crop were sown in each pot and then thinned to three seedlings per pot after 7 days. The carrot crop was further thinned to one seedling per pot after 12 days. Four replicates were conducted for each treatment (*i.e*., the soil sample with different total As concentrations). Pots were placed randomly in a phytotron (day/night = 25 °C/20 °C) with natural light.

Chemical fertilizers were applied to each pot as (NH_2_)_2_CO, Ca(H_2_PO_4_)_2_·H_2_O, and KCl. For carrot, 19.8, 17.5, and 8.2 mg/kg soil of N, P, and K were applied before planting. Thirty days after germination, chemical fertilizer (N:P:K = 59.4:6.5:32.8 mg/kg soil) was reapplied to the soils. Additional chemical fertilizers (N:P:K = 30.8:3.3:50.2 mg/kg soil) were applied after 70 days of germination. For garland chrysanthemum, chemical fertilizers were applied as 37.5 mg N/kg soil, 24 mg P/kg soil, and 29.1 mg K/kg soil. After 20 days of germination, additional fertilizer (37.5 mg N/kg soil and 29.1 mg K/kg soil) was applied to the soils. For loose head lettuce and loose leaf lettuce, 60.0, 43.7, and 49.8 mg/kg soil of N, P, and K, respectively, were applied to the soils. Additional fertilizer (60.0 mg N/kg soil and 49.8 mg K/kg soil) was applied to the soils after 20 days of germination.

### 2.6. Harvest and Plant Analysis

Edible parts of the carrot (the skin was not peeled), garland chrysanthemum, loose head lettuce, and loose leaf lettuce were harvested after the crop samples had grown for 90, 60, 40, and 40 days, respectively. Samples were first washed with tap water and then deionized water several times to remove adhered soil particles and dust. The crop samples were oven-dried to constant weight at 70 °C for 3 days. The dried samples were weighed and then powdered homogenously for analysis. The total arsenic concentrations of crop samples were analyzed with the HNO_3_/H_2_O_2_ digestion method, and the method detection limit of As was 0.040 mg/kg DW. NIST SRM 1573a tomato leaves (certified As concentration: 0.112 ± 0.004 mg/kg) were used as the CRM for quality control of the laboratory analysis. The average total As recovery percentage of the CRM by the HNO_3_/H_2_O_2_ digestion method was 91% ± 8%.

### 2.7. Statistical Analysis

Analysis of variance and regression analysis were conducted by using the statistical software R. Mean comparisons were carried out with Tukey’s honest significant difference (HSD) test at a significance level at *p* = 0.05. Linear regression was used to determine the relationships between soil As bioavailability with soil testing and As in different vegetables.

## 3. Results

### 3.1. Effects of Soil As Concentrations on the Yields and As Accumulation

The soil samples for the pot experiments were slightly acidic (pH 4.45–6.43), with high soil organic matter content (2%–5%) and mostly fine soil texture. Table 1 shows the As concentrations of the soil samples (Ck, L, M, H, and 2H) and the corresponding crop yields and As concentration in the edible parts of the carrot, garland chrysanthemum, and loose head lettuce samples. 

**Table 1:** As concentrations in soil samples and corresponding crop yields and As concentrations in edible parts of different crops.

Soil Sample ^a^	Soil As Conc. ^b^		Crop ^b^		
	Total Conc.	Bioavailable Conc. ^c^	Yield	Total As Conc.	BCF ^d^
	mg/kg		g/pot	mg/kg DW	
Carrot					
Ck	20.1 d	0.41 d	9.02 a	nd	--
L	121 c	2.75 c	9.35 a	0.078 a	0.028
M	200 b	5.13 b	10.0 a	0.102 a	0.020
H	278 a	6.46 a	9.63 a	0.155 a	0.024
Garland Chrysanthemum					
Ck	21.4 d	0.35 d	2.19 a	nd	--
L	124 c	2.67 c	2.25 a	0.374 b	0.140
M	194 b	5.10 b	2.31 a	0.784 a	0.154
H	265 a	6.58 a	2.97 a	0.825 a	0.125
Loose Head Lettuce					
Ck	20.7 d	0.50 d	2.11 b	nd	--
L	123 c	3.26 c	2.05 b	0.195 b	0.060
M	197 b	5.38 b	1.30 b	0.258 b	0.048
2H	453 a	8.60 a	3.74 a	0.515 a	0.060

^a^ Ck, L, M, H, and 2H denote intrinsic As-contaminated soils with different levels of total As, control (Ck), low (L), M (medium), H (high), and 2H (very high). nd: not detectable (lower than the method detection limit for As in crops: 0.040 mg/kg DW); ^b^ Data are the mean value of four replicates. Data marked with different letters in each column of each crop indicate a significant difference at *p =* 0.05 according to Tukey’s HSD; ^c^ Bioavailable As in soil was extracted with 0.5 M NaHCO_3_; ^d^ BCF (bioconcentration factor) = total As concentration in crops/bioavailable As concentration in soil.

Regardless of the total As concentration in soils, no toxicity syndrome as described in previous studies [5,6] was found in any of the three vegetables during the growth period. In addition, there was no significant difference among the yields of the edible part of carrots grown in soils with different levels of total As. Similar results were also found for garland chrysanthemum and loose head lettuce except for 2H. These results show that the growth and yields of vegetable crops were not affected by the As-contaminated soils on the Guandu Plain. This is a good complement to the incomplete findings of a previous field survey that had reported that there was no toxic syndrome of vegetable crops cultivated in the As-contaminated soils on the Guandu Plain [24].

The As concentrations in the carrot samples were all lower than 0.2 mg/kg DW (equals 0.034 mg/kg FW, based on the average water content, 83%), and there was no significant difference among the different soil As concentrations (Table 1). Among the three vegetables cultivated in this study, the As concentration in the edible part of the carrot samples was the lowest; however, the As concentrations in the edible parts of three vegetables increased as the total As concentration increased in the soils (Table 1). The arsenic level in loose head lettuce was not as high as in garland chrysanthemum even in the 2H treatment. The highest average As concentrations in garland chrysanthemum and loose head lettuce were 0.825 mg/kg DW (equals 0.074 mg/kg FW, based on the average water content, 91%) and 0.515 mg/kg DW (equals 0.021 mg/kg FW, based on the average water content, 96%), respectively.

### 3.2. Effects of Changing the Bioavailability of As on Yields and Accumulation of As

Not only *in situ* As-contaminated soils sampled in the fields from the Guandu Plain but also artificial As-contaminated soils with higher As bioavailability were used to grow loose leaf lettuce. We expected that the artificial As-contaminated soils would have negative effects on the growth of loose leaf lettuce. However, no toxicity syndrome was found in the cultivated loose leaf lettuce samples during the pot experiment. In addition, there were no significant differences in the yields of loose leaf lettuce cultivated in different treatments, although the highest bioavailable As concentration in the artificial As-contaminated soil (Ck2 + 150) was 2.6 times that of 2H (Table 2).

The bioconcentration factor (BCF = total As concentration in crop/bioavailable As concentration in soil) was used in this study to assess the As accumulation capacity of different vegetables. Excluding Ck and Ck2, the BCF of carrot, garland chrysanthemum, loose head lettuce, and loose leaf lettuce was 0.024 ± 0.004, 0.140 ± 0.014, 0.056 ± 0.007, and 0.054 ± 0.013, respectively. The experimental results showed that the As accumulation capacity of the four vegetables was in the following order: garland chrysanthemum > loose head lettuce > loose leaf lettuce > carrot. The As concentration in the edible parts of the leaf vegetable crops was higher than that of the root vegetable crops. Results of this study are in agreement with previous studies conducted in As-contaminated fields in Chile and Bangladesh [30,31]. The highest average As concentrations in loose leaf lettuce reached 0.970 mg/kg DW (equals 0.097 mg/kg FW, based on the average water content, 90%). The As concentrations in the edible part of loose leaf lettuce clearly increased as the bioavailable As concentrations in the soils increased (Table 2). This implied that if the bioavailability of As in soils on the Guandu Plain increased (probably due to the addition of P or lime materials, unpublished data), As levels in the cultivated vegetable crops would increase and thus produce potential risks to human health.

**Table 2:** Total and bioavailable As concentrations of nine studied soils and total As in the edible part of loose leaf lettuce.

Soil Sample ^a^	Soil As Conc. ^b^		Crop ^b^		
	Total Conc.	Bioavailable Conc. ^c^	Yield	Total As Conc.	BCF ^d^
	mg/kg		g/pot	mg/kg DW	
Ck2	18.9 h	0.33 g	3.13 a	0.077 f	0.233
L2	139 de	3.40 f	2.98 a	0.259 e	0.076
M2	239 b	6.60 e	2.34 a	0.311 e	0.047
H2	475 a	8.93 d	3.56 a	0.362 de	0.041
Ck2+30	50.2 g	4.51 f	2.66 a	0.323 e	0.072
Ck2+60	87.1 f	8.60 d	2.63 a	0.489 d	0.057
Ck2+90	120 e	13.7 c	2.53 a	0.625 c	0.046
Ck2+120	151 cd	16.2 b	2.82 a	0.792 b	0.049
Ck2+150	172 c	22.2 a	2.85 a	0.970 a	0.044

^a^ Ck2, L2, M2, and H2 denote the intrinsic As-contaminated soils with different levels of total As. Ck2 + 30, Ck2 + 60; Ck2 + 90, Ck2 + 120, and Ck2 + 150 denote the artificial As-contaminated soils spiked with different levels of As (mg/kg); ^b ^Data are the mean of four replicates. Data marked with different letters in each column indicate a significant difference at *p* = 0.05 according to Tukey’s HSD; ^c^ Bioavailable As in soil was extracted with 0.5 M NaHCO_3_; ^d^ BCF (Bioconcentration factor) = Total As concentration in crop/bioavailable As concentration in soil.

### 3.3. Comparison of Different Soil Testing Methods for As Crop Predictions

**Figure 1:**
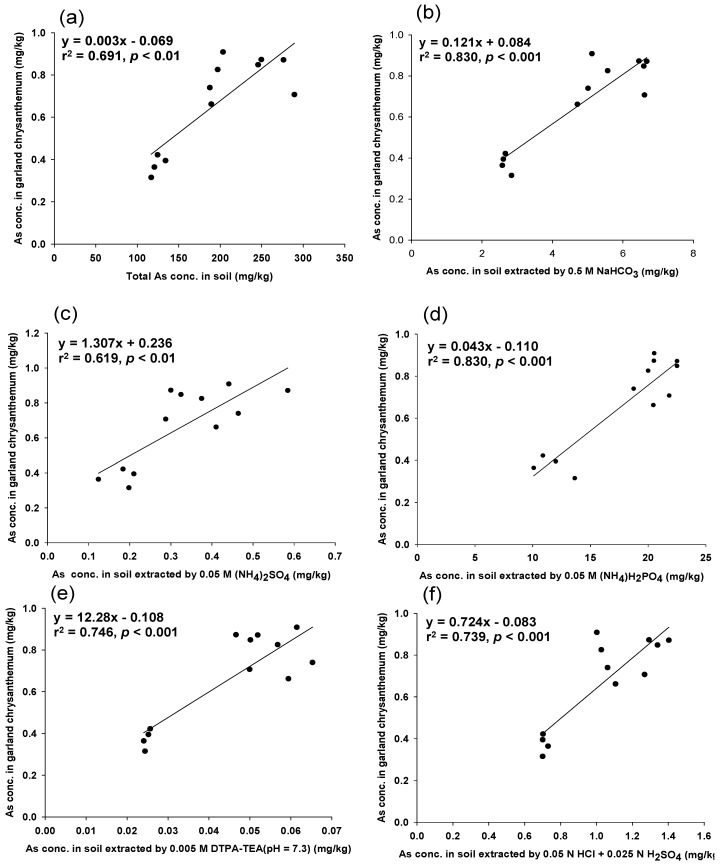
The relationship between arsenic concentrations in garland chrysanthemum and in soils extracted with (**a**) HNO_3_/H_2_O_2_; (**b**) 0.5 M NaHCO_3_; (**c**) 0.05 M (NH_4_)_2_SO_4_; (**d**) 0.05 M (NH_4_)H_2_PO_4_; (**e**) 0.005 M DTPA-TEA(pH 7.3); and (**f**) 0.05 N HCl + 0.025 N H_2_SO_4_.

Figure 1 shows the linear relationships between the As levels extracted with different methods in garland chrysanthemum and in soils (*p* < 0.01 or *p* < 0.001). Due to the higher correlation coefficient value, the two soil testing methods extracted with NaHCO_3_ or (NH_4_)H_2_PO_4_ were selected to further assess their ability to predict As levels in loose head lettuce. 

**Figure 2:**
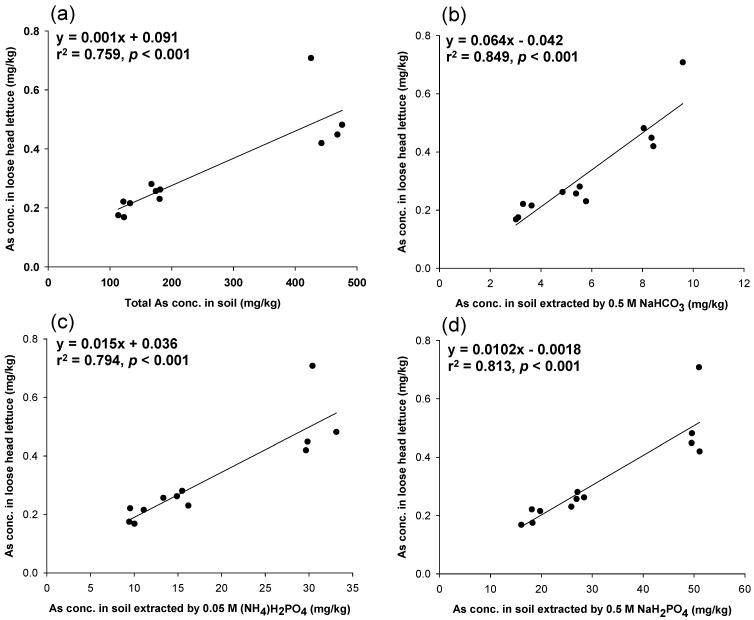
The relationship between As concentrations in loose head lettuce and in soils extracted with (**a**) HNO_3_/H_2_O_2_; (**b**) 0.5 M NaHCO_3_; (**c**) 0.05 M (NH_4_)H_2_PO_4_; and (**d**) 0.5 M NaH_2_PO_4_.

**Figure 3:**
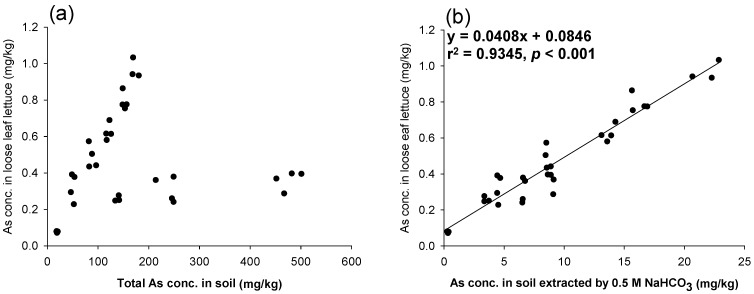
The relationship between As concentrations in loose leaf lettuce and (**a**) total As in soils; (**b**) As in soil extracted with 0.5 M NaHCO_3_.

Figure 2 also presents the linear relationships between the As levels in loose head lettuce and in soils extracted with different soil testing methods (*p* < 0.001). The soil testing method that used 0.5 M NaHCO_3_ not only showed the best prediction but was also the simplest and quickest soil testing method operated in the laboratory. Therefore, this method was used to assess its ability to predict As levels in loose leaf lettuce cultivated in intrinsic and artificial As-contaminated soils. In this case, the total As concentrations in soils were not a good indicator for predicting As levels in the edible parts of loose leaf lettuce, as shown in Figure 3a. However, the 0.5 M NaHCO_3_ predicted the As concentrations in the edible parts of loose leaf lettuce (Figure 3b). This study demonstrated that the simplest and fastest soil testing method, 0.5 M NaHCO_3_, could be used to estimate As concentrations in vegetables before cultivation. The pH of the 0.5 M NaHCO_3_ in this study was not adjusted to pH 8.5 as in previous studies [16,17]. This also saves time in preparing the reagent. However, because the data points resided in two clusters, this strengthened the linear relationships presented in Figure 1 and Figure 2a–d.

## 4. Discussion

Sheppard [2] reported that the detrimental effects of soil As on plants depend on the types of soil. He concluded that the average phytotoxicity threshold of As in sandy soils and in clayey soils is 40 and 200 mg/kg, respectively. The total As concentration in the 2H soil in this study was more than two times or higher than the reported average phytotoxicity threshold for clayey soils, but loose head lettuce still grew well (Table 1). This result perhaps was due to the very high amounts of amorphous Fe and Al oxides in the study soils and the aging effect reduced the bioavailability of As in high As-contaminated soils of the Guandu Plain (unpublished data). Beni *et al.* [32] cultivated lettuce in sandy soil irrigated with water that had different As levels (19–104 μg/L). The results also showed that there was no difference in lettuce biomass between treatments. The loose leaf lettuce crops seem to resist As toxicity even when they are grown in artificial As-contaminated soils with higher bioavailability.

There are no regulatory criteria for As in foodstuffs in Taiwan. Compared with the regulation standard of As for food vegetables in different countries reported in the literature(Table 3), the As levels of the studied vegetables grown in As-contaminated soils on the Guandu Plain were relatively low and seemed to be safe for consumers near the Guandu Plain. However, the following investigation in terms of human intake showed the potential risks to human health.

**Table 3:** Regulation limits of As concentration in cereals or food crops established by different countries.

Country	Item regulated	Statutory limits ^a^	Reference
Canada	food crops	1 mg/kg FW	[33]
United Kingdom	food in sale	1 mg/kg FW	[34]
China	rice	0.15 mg/kg^ b^	[35]
Australia	cereals	1 mg/kg FW	[36]
New Zealand	cereals	1 mg/kg FW	[36]
Germany	cereals	1 mg/kg FW	[37]
India	cereals	1 mg/kg FW	[37]
The Netherlands	cereals	1 mg/kg FW	[37]

^a^ FW: fresh weight; DW: dried weight; ^b^ For inorganic As.

To understand the potential risks to human health of vegetables cultivated in the As-contaminated fields of the Guandu Plain, we compared the experimental results of this study with other studies reported in the literature (Table 4). The As concentration in the crop samples in our study was apparently higher than those of the samples in markets. In foreign As-contaminated fields, some vegetable crops can accumulate relatively high amounts of As (>1 mg/kg DW), even when they were cultivated in slightly As-contaminated soils [14,26]. In contrast, the As levels in the crop samples of this study grown in intrinsic highly As-contaminated soils were lower than 0.9 mg/kg DW. Compared with the data published in the literature, it is interesting to find that the As levels of vegetable crops of this study were lower than those of foreign As-contaminated area; even the As concentrations in the soils on the Guandu Plain were apparently high. This indicated that the bioavailability of As in the soils of the Guandu Plain is relatively low even when the total As levels in soils are very high (120–460 mg/kg).

**Table 4:** As concentrations in vegetables and soils reported in literature and in this study.

Study Area	As Conc. in the Vegetable (mg/kg DW)			Total As Conc. in Soil (mg/kg)	Reference
	Mean	Range	*n*		
Markets					
Europe	0.0242	<0.005–0.087	24	--	[38]
Europe	0.0545	<0.005–0.54	68	--	[38]
Canada	0.007 ^a^	--	262	--	[39]
U.K.	0.005 ^a^	--	60	--	[40]
The Netherlands ^b^	0.001–0.189 ^a^	0.0001–0.544	39–94	0.1–110	[41]
The Netherlands ^c^	0.004–0.022 ^a^	0.005–0.014	50–100	0.1–110	[41]
As-Contaminated Area					
Bangladesh	--	0.019–0.489	>15	13.3	[42]
Bangladesh	--	0.007–1.53	11	7.3–27	[14]
West Bengal	--	<0.04–0.69	142	3.3–32	[13]
Spain	--	0.3–1.25	57	9–36	[26]
Bangladesh	0.333	0.019–2.334	39	--	[10]
Bangladesh	0.34	<0.04–1.93	94	--	[31]
This Study					
Guandu Plain, Taiwan	0.332	<0.040–0.873	64	18–501	

^a^ The concentration is based on fresh weight; ^b^ Greenhouse crops; ^c^ Vegetables in the open; --: data not shown.

Since As is a highly toxic and carcinogenic agent [1], it is possible that a stringent limit for As in foodstuffs will be imposed in Taiwan. In fact, the European Food Safety Authority (ESFA) reported that the PTWI of inorganic As, which is 15 µg/kg body weight established by the Joint FAO/WHO Expert Committee on Food Additives (JECFA), is no longer appropriate and has recommended the value be reduced [43].Using the technique for predicting the As level of vegetables, local governments can help farmers ensure their production will not exceed the As standard. We recommend the government regularly monitor the As concentration of vegetables and the corresponding As bioavailability in soils on the Guandu Plain with a simple soil testing method using 0.5 M NaHCO_3_ as the reagent.

Although the As concentrations in the edible parts of studied vegetables in the Guandu Plain (Table 1 and Table 2) were much lower than the standards reported for different countries in the literature (Table 3), As intake by humans via vegetable crop consumption may still pose a risk for long-term consumption due to the high carcinogenicity of As. The food safety of vegetables should be reevaluated based on other established indexes, such as the PTWI of inorganic As or the As standard in drinking water. The PTWI guideline for As and drinking water standards are based on the inorganic As concentration rather than the total As concentration. Although this study determined only the total As concentrations in vegetable, studies in the literature have reported that inorganic As is about 70%–100% of the total As concentrations in vegetables [15,30,31,44]. To ensure the food safety of vegetable crops, it is reasonable to assume that 100% of total As in vegetable is inorganic As.

The PTWI of inorganic As was 15 µg/kg body weight. The margin between the PTWI and intakes reported in epidemiological studies to produce toxic effects was narrow [1]. Assuming the average body weight of a Taiwanese adult is 60 kg, the tolerance daily As intake for an adult is 129 µg (15 µg/kg body weight/wk ÷ 7 days/wk × 60 kg). According to official 2010 Taiwanese statistics, the average amount of daily vegetable consumption per person is 286 g fresh weight. Assuming the average water content of vegetable crops is 90%, from these values we can calculate that if the levels of As in vegetable is higher than 4.50 mg/kg DW, the As intake via vegetables will be higher than the PTWI.

In Taiwan, U.S., and WHO, the established standard of inorganic As in drinking water is 10 µg/L. Under the assumption mentioned and assuming the amount of water an adult drinks every day is 2 L, we calculated that if the level of As in vegetables reached 0.699 mg/kg DW, the As intake via consuming vegetables is the same as the As intake via drinking water that contains 10 µg As/L.

**Figure 4:**
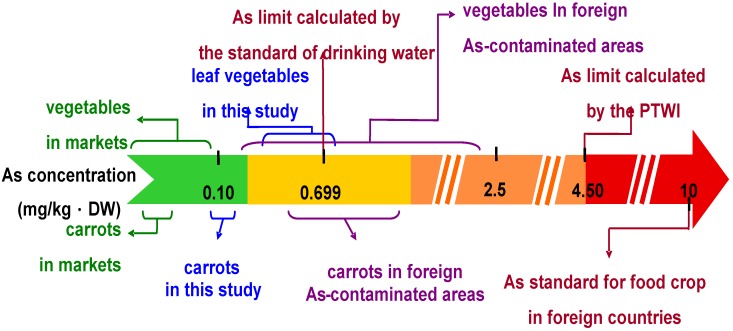
Summary of As concentrations in vegetable crops in this study, data in the literature, As limits calculated with the key indexes, and the regulation standard for foodstuffs.

In Figure 4, the degree of As contamination in the studied vegetable crops is shown by comparing the As levels with data in the literature and As limits derived from key indexes. The As levels in the vegetable crops produced in As-contaminated fields on the Guandu Plain were much lower than the As limit calculated from the PTWI or the As standard for food in many countries reported in the literature. However, the levels were still higher than the As levels in vegetables collected from general markets and sometimes higher than the As limit calculated from the standard for drinking water, even though they were lower than As levels in vegetables produced in foreign As-contaminated areas. Accordingly, compared with vegetables from general markets, long-term consumption of vegetable crops produced from As-contaminated fields on the Guandu Plain may result in higher As intake in the human body.

Although the As bioavailability in the highly As-contaminated soils on the Guandu Plain is quite low, As accumulation in vegetable crops also depends on plant species and agronomic managements [30,45]. To protect human health, a comprehensive survey of As levels in various vegetables cultivated in the Guandu Plain, the distribution of the vegetables in local markets, and the corresponding food safety assessment is necessary.

## 5. Conclusions

No phytotoxic effects of As on vegetables were found during pot experiments when vegetable crops were grown in *in situ* collected Guandu Plain soils or artificially spiked As-contaminated soils. Although the arsenic levels in vegetable crops grown in As-contaminated fields on the Guandu Plain were low, long-term consumption may result in higher As intake in the human body.
